# Pediatric Dental Emergencies during the COVID-19 Pandemic in Romania: A Retrospective Study

**DOI:** 10.3390/children10050807

**Published:** 2023-04-29

**Authors:** Abel Emanuel Moca, Raluca Iurcov, Gabriela Ciavoi, Rahela Tabita Moca, Lucian Roman Șipoș

**Affiliations:** 1 Department of Dentistry, Faculty of Medicine and Pharmacy, University of Oradea, 10 Piața 1 Decembrie Street, 410073 Oradea, Romania; abelmoca@yahoo.com (A.E.M.); gciavoi@uoradea.ro (G.C.); luci_sip@yahoo.com (L.R.Ș.); 2 Doctoral School of Biomedical Sciences, University of Oradea, 1 Universității Street, 410087 Oradea, Romania; rahelamoca@gmail.com

**Keywords:** pediatric dental emergencies, Romania, pandemic, COVID-19

## Abstract

Pediatric dental emergencies can occur as a result of untreated dental caries, or can be caused by trauma or periodontal issues. The lockdown imposed during the COVID-19 pandemic reduced the number of centers authorized to deliver dental services in Romania, with only a small number of dentists delivering dental emergency treatments. The aim of this study was to investigate the demographic characteristics of children and adolescent patients who were treated in the dental emergency department of Oradea, Romania and to compare the patients who were treated in the dental emergency department in the pre-lockdown (2019), lockdown (2020) and post-lockdown year (2021). All patients who were treated in the dental emergency department were included in the study except for adults and medical files that did not contain all relevant information. Several variables were investigated (age, gender, living environment, location of affected teeth, type of emergency). In 2019, 257 children and adolescents were treated, in 2020, 198, and in 2021, 136. Most patients were aged 7–12 years in all investigated years (2019—47.9%; 2020—50.5%; 2021—43.4%), and the most affected teeth were located in the lower posterior arch (2019—53.3%; 2020—53%; 2021—48.5%). The most frequent emergencies were pulpitis (2019—40.5%; 2020—43.9%) and acute apical periodontitis (2021—42.6%). It was observed that in 2019, patients aged between 0 and 6 years were more frequently associated with trauma (*p* < 0.001), and in 2019 and 2020, patients aged between 7 and 12 years were more frequently associated with periodontal emergencies (*p* < 0.001). In 2020, patients from rural areas were more frequently associated with pulpitis (*p* = 0.025), and in 2021, patients from rural areas were more frequently associated with pulpitis or acute apical periodontitis, and patients from urban areas were associated more frequently with periodontal emergencies (*p* = 0.042). Pediatric patients with ages between 7 and 12 years old, who lived in an urban environment were most affected. Teeth located in the lower and upper posterior dental arches were most affected, and pulpitis or acute apical periodontitis were the most common pathologies.

## 1. Introduction

Dental caries and periodontal diseases represent a global public health issue [1], which can affect people of any age [2]. Dental caries is a multifactorial disease [3] and is the result of the interaction between the poor quality of the tooth structure, cariogenic microorganisms, a high sugar diet, and other environmental factors [4]. Dental caries is also the most prevalent oral disease [5]. If left untreated, dental caries can lead to complications such as pulpitis, pulp necrosis, and abscesses [6], possibly even leading to the loss of the affected tooth [7]. Pulpitis, acute apical periodontitis, abscesses, but also trauma, are dental emergencies that require rapid intervention [8].

Children and adolescents are the population groups affected by dental caries to varying degrees [9]. According to the European Union, the average incidence of dental caries in children under the age of 5 years is reported to be 37.2% [10] and has reached 77% in adolescents aged 12–19 years [11]. Untreated dental caries has a global high prevalence and can rapidly become dental emergencies [12]. Dental traumas represent dental emergencies and have a higher prevalence among children and adolescents [13]. In Romania, the prevalence of dental caries among children and adolescents is very high, with different authors reporting a prevalence varying from 85.2% in children aged between 6 and 8 years [14], up to 95.5% in adolescents aged up to 19 years [15]. The prevalence of dental trauma was reported as being 24.5% in pediatric patients in Romania [16].

During the lockdown imposed due to the COVID-19 pandemic, in addition to the various restrictions and policies adopted to limit the spread of the SARS-CoV-2 virus, a quarantine was installed, and as a result, the operation of dental offices was suspended [17]. In Romania, the lockdown period began on 21 March 2020 and ended on 15 May 2020 [18]. During this time, the only dental treatments that could be performed were dental emergencies, which could only be managed in centers that were authorized by the government [19]. The following pathologies were considered dental emergencies during the lockdown period: post-extraction hemorrhages, acute pulpitis, acute apical periodontitis, pericoronitis, post-extraction alveolitis, abscesses, cellulitis, mandibular fractures, dislocations of the temporomandibular joint, and ulceronecrotic gingivostomatitis [20].

There is a general high prevalence of dental caries among children and adolescents in Romania [14,15] due to various factors, including relatively rare dental visits among pediatric patients [21], and rather than for preventive reasons, children’s first dental visits are overwhelmingly due to dental pain [22]. However, it was postulated that the number of children and adolescents who needed dental emergency treatment was higher during the COVID-19 pandemic than in other years, because unfortunately, most dental offices were closed during the COVID-19 lockdown [17]. This aspect requires attention because the burn-out rate among the medical staff increased during the pandemic [23], and the overloading of emergency dental departments could subsequently have negative effects on the mental and physical health of the dental staff.

In light of the above, the aim of this study was to investigate the demographic characteristics of children and adolescent patients who were treated in the dental emergency department of Oradea, Romania. Another aim was to compare the patients who were treated in the dental emergency department between 21 March 2020 and 15 May 2020 (lockdown), with those who were treated between 21 March 2019 and 15 May 2019 (pre-lockdown), or between 21 March 2021 and 15 May 2021 (post-lockdown).

## 2. Materials and Methods

### 2.1. Ethical Considerations

The research was carried out with respect to the principles stated in the 2008 Declaration of Helsinki and in the amendments that were later added. Considering that the sample was composed mainly of underaged patients, at the time of admittance into the dental emergency department, the signed consent of the parents or legal guardians was requested so that the data obtained could be used anonymously in future studies. Patients who were 18 years old provided their consent for the anonymous use of their data. The study was approved by the Ethics Committee of the Oradea County Emergency Clinical Hospital (IRB No. 22143 from 6 July 2022).

### 2.2. Data Collection and Participants

The retrospective study was carried out by analyzing the medical files of children and adolescent patients who were registered at the dental emergency department of the Oradea County Emergency Clinical Hospital. This department is located in the building of the Faculty of Medicine and Pharmacy in Oradea and offers continuous treatments of dental emergencies for both children and adult patients. Emergency treatments are provided free of charge. At the beginning of the lockdown, and due to the onset of the COVID-19 pandemic, this was the only medical center that treated dental emergencies in the city of Oradea and in the Bihor county. The medical files of patients who came for the treatment of dental emergencies in the following time periods were analyzed: 21 March 2019–15 May 2019 (pre-lockdown), 21 March 2020–15 May 2020 (lockdown) and 21 March 2021–15 May 2021 (post-lockdown).

All medical records registered during the mentioned time periods were initially read and analyzed. The medical records that belonged to patients aged 19 years or older were later excluded, as well as the medical records that did not contain sufficient information regarding the variables investigated in this study. In addition, the medical files of patients who came to the dental emergency department but who had emergencies related to another medical specialty or had no dental emergencies were excluded from the study.

The following variables were investigated in this study: gender (girls, boys); age group (0–6 years, 7–12 years, 13–18 years); living environment (rural—people who live in villages, urban—people who live in small or large cities); location of affected teeth (upper anterior, upper posterior, lower anterior, lower posterior); and the type of dental emergency (pulpitis, acute apical periodontitis, abscess, trauma, periodontal emergencies). These variables were selected from the medical files and were organized in a Microsoft Excel document (Microsoft, Redmond, WA, USA).

Two authors independently verified all medical records. This was performed in order to avoid bias.

### 2.3. Statistical Analysis

Microsoft Office Excel/Word 2013 (Microsoft, Redmond, WA, USA) and IBM SPSS Statistics 25 (IBM, Chicago, IL, USA) were used for the statistical analysis. Qualitative variables were reported as absolutes or as percentages, and the Fisher’s exact test was used to compare them. The data from the contingency tables were detailed using Z-tests with the Bonferroni correction. A value of *p* < 0.05 was necessary in order to consider that the results are statistically significant.

## 3. Results

A total number of 1783 medical files belonging to adult and pediatric patients were registered in the studied periods of time, out of which, 569 were registered between 21 March and 15 May 2019, 704 between 21 March and 15 May 2020, and 510 between 21 March and 15 May 2021. After applying the exclusion criteria, 257 medical files that were recorded in 2019 (45.2%), 198 that were recorded in 2020 (28.1%), and 136 that were recorded in 2021 (26.7%) remained in the study.

### 3.1. Gender, Age, Living Environment, and Location of the Affected Tooth

The highest number of patients was registered in 2019 (*n* = 257) in the pre-lockdown period, and the lowest number was registered in 2021 (*n* = 136) in the post-lockdown period. In 2020, a total of 198 patients were treated in the department.

In 2019, 46.7% of patients were girls (*n* = 120) and 53.3% were boys (*n* = 137). In 2020, 52.5% of the patients were girls (*n* = 104) and 47.5% were boys (*n* = 94), while in 2021, 48.5% were girls (*n* = 66) and 51.5% were boys (*n* = 70). In all three years, the vast majority of patients were made up of patients who lived in an urban environment. In 2019, 52.9% were from the urban environment (*n* = 136) and 47.1% were from the rural environment (*n* = 121). In 2020, 55.6% of patients were from the urban environment (*n* = 110) and 44.4% were from the rural environment (*n* = 88). In 2021, 53.7% of patients were from the urban environment (*n* = 73) and 46.3% were from the rural environment (*n* = 63).

Regarding patients’ age, in all the three years, most patients were between 7 and 12 years old. Thus, in 2019, 47.9% (*n* = 123) of the patients were aged between 7 and 12 years, in 2020, 50.5% (*n* = 100), and in 2021, 43.4% (*n* = 59). The distribution of the different age groups is shown in Table 1.

The location of the most frequently affected and treated teeth in the dental emergency department is provided in Table 2. In all years, the most frequently affected teeth were located in the lower posterior arch (2019—53.3%, *n* = 137; 2020—53%, *n* = 105; 2021—48.5%, *n* = 66), and the least affected were located in the lower anterior arch (2019—3.9%, *n* = 10; 2020—1.5%, *n*= 3; 2021—2.9%, *n* = 4).

The data in Table 3 show the distribution of patients from 2019, 2020, and 2021 in relation to the location of the treated teeth, as well as gender, age, and living environment. According to Fisher’s exact tests, the associations between the location of teeth and gender (2019—*p* = 0.572; 2020—*p* = 0.949; 2021—*p* = 0.358) or living environment (2019—*p* = 0.325; 2020—*p* = 0.185; 2021—*p* = 0.825) were not statistically significant, but a significant association was observed between age and teeth location in 2019 (*p* = 0.002) and 2020 (*p* = 0.029). In 2019 and 2020, the Z-tests with the Bonferroni correction showed that patients aged 0–6 years were more frequently associated with affected teeth located in the upper anterior arch (2019—52.8%; 2020—35%) than teeth located in the upper posterior arch (2019—17.6%; 2020—17.1%) or lower posterior arch (2019—16.8%; 2020—14.3%).

### 3.2. Type of Emergency

**Figure 1:**
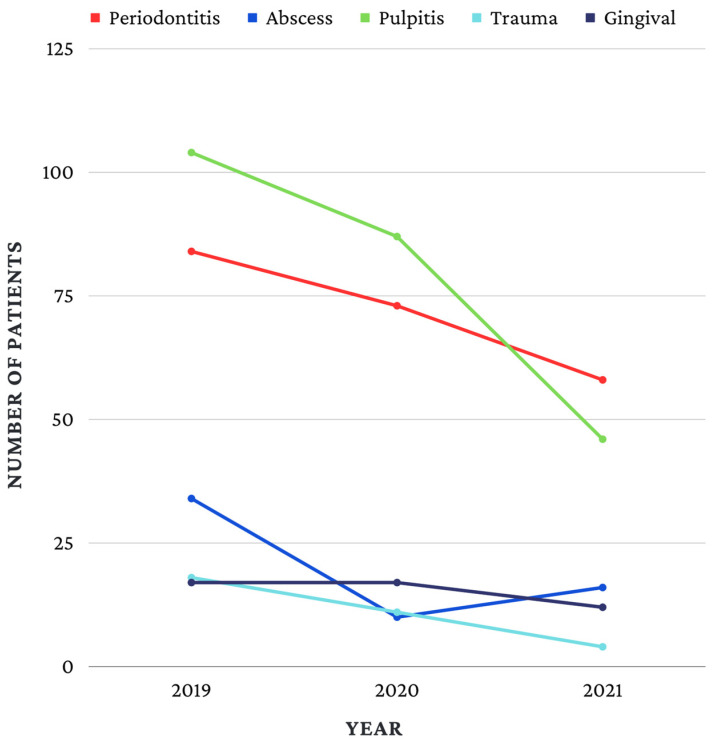
Types of emergency in 2019, 2020, 2021.

In 2019 and 2020, the most frequent emergencies were pulpitis (2019—40.5%, *n* = 104; 2020—43.9%, *n* = 87) and acute apical periodontitis (2019—32.7%, *n* = 84; 2020—36.9 %, *n* = 73). In 2021, the order between the two emergencies was reversed, so that the most frequent pathology in 2021 was acute apical periodontitis (42.6%, *n* = 58), followed by pulpitis (33.8%, *n* = 46) (Figure 1). The data in Table 4 show the evolution between the three investigated years related to the identified types of emergency. The results showed that the differences between the frequency of emergencies and age were significant (*p* = 0.031), and Z-tests with the Bonferroni correction showed that abscesses were more frequent in 2019 (13.2%) than in 2020 (5.1%).

The data in Table 5 represent the distribution of patients from 2019, 2020, and 2021 in relation to the type of emergency, gender, age, and living environment. According to Fisher’s exact tests, the associations between the type of emergency and gender were not statistically significant in any of the investigated years (2019—*p* = 0.110; 2020—*p* = 0.713; 2021—*p* = 0.467). However, a significant association between age and type of emergency was observed in all three years (2019, 2020, 2021—*p* < 0.001), and subsequent tests showed that in 2019, patients aged 0–6 years were associated more frequently with trauma, and patients aged 7–12 years were more frequently associated with periodontal emergencies. In 2020, patients aged 7–12 years were more frequently associated with periodontal emergencies or apical periodontitis than with pulpitis, while patients aged 13–18 years were more frequently associated with acute pulpitis than with acute apical periodontitis. In 2021, patients aged between 0 and 6 years were more frequently associated with abscesses, and patients aged between 13 and 18 years were more frequently associated with pulpitis or acute apical periodontitis.

Regarding the living environment, significant associations were identified between the type of emergency and the living environment in 2020 and 2021. In 2020, patients from rural areas were more frequently associated with pulpitis than with other emergencies, and patients from urban areas were less often associated with pulpitis than other emergencies. In 2021, patients from rural areas were more frequently associated with pulpitis or acute apical periodontitis than with periodontal emergencies, and patients from urban areas were more frequently associated with periodontal emergencies than with acute apical periodontitis or pulpitis (Table 5).

## 4. Discussion

The COVID-19 pandemic and the subsequent lockdown impacted many areas of everyday life [23], with dentistry being one of the areas affected [17]. Considering the neglectful attitude toward oral health and the high prevalence of caries among the pediatric population in Romania [14,15], it was suspected that during the 2020 lockdown, the number of pediatric patients who were treated in the dental emergency department was higher than during the pre-lockdown period of 2019 or the post-lockdown period of 2021. Knowing these aspects is important not only for monitoring the oral health of Romanian children and adolescents, but also for monitoring the level of physical burden and mental overload of the medical staff working in the dental emergency department. In addition, it is important to highlight the fact that dental pediatric patients represent an additional challenge and require various behavior management strategies [24].

The results of our study showed that the highest number of children and adolescents who were treated for a dental emergency was recorded in 2019, and the lowest was recorded in 2021. In 2020, of all the patients who were treated for a dental emergency, 28.1% were children and adolescents. However, the largest number of adult patients and children who were treated in the dental emergency department was recorded in 2020, during the lockdown period. The high percentage of children treated in 2019 may be due to the neglect of the prevention and treatment of simple caries in children [25], with dental visits being most often prompted by acute dental pain [26]. The number of patients was only slightly lower in 2020, compared to 2019, a fact that underlines that which was previously mentioned. The difference between the number of adults and children treated in 2020 is higher. This is most likely caused by the suspension of the operation of dental offices during the lockdown period [17], and so all dental emergencies had to be treated in authorized centers [19]. The total number of adults and children is similar in 2019 and 2021, but the number of children and adolescents treated in 2021 is considerably lower than that of children and adolescents treated in 2019. It can be considered that this fact is caused by an increase in parents’ vigilance and interest in children’s oral health, even if the situation was far from ideal [27].

In the three years investigated, most of the pediatric patients came from the urban environment. This can be caused, on the one hand, by the fact that the dental emergency department is located in an urban area, and on the other hand by the fact that in Romania, the majority of the population lives in the urban environment [28]. The focus on addressing oral health in the rural population is very limited [29], and access to dental services is restricted by factors such as the small number of rural dental offices or the high costs of dental treatments [30]. Other authors reported a lower share of preventive interventions in the rural population, and therefore a higher share of curative treatments [31]. The patients were also distributed according to age. The distribution into three age groups (0–6 years, 7–12 years, 13–18 years) was preferred as it is similar to other studies that investigated the impact of the COVID-19 pandemic on children’s oral health [32], and also because it comprises the preschool (0–6 years), school (7–12 years) and adolescence (13–18 years) development phases [33]. Most patients were distributed in the 7–12 years age group in all three years. The same predominance of patients in the 7–12 age group was reported by other authors [32]. It was observed that in 2019 and 2020, patients in the 0–6 years age group were more frequently associated with treatments for affected teeth in the upper anterior arch. Pathologies in this area and at this age occur either as a result of dental trauma or as a result of the rapid evolution of ECC [34]. However, other authors reported a higher prevalence of dental caries in deciduous maxillary and mandibular molars [35]. Similarly, in this study, teeth located in the upper and lower posterior arches were most frequently affected. This is in line with several studies that have reported an increased incidence of caries in first permanent molars [36,37]. Contrary to our findings, there are authors who reported a higher incidence of caries in the upper arch than in the lower arch [38], but our results are consistent with the results obtained in a previously published radiographic study in which first permanent molars were investigated, and in which superficial, medium, or deep caries prevailed in the lower arch [39].

The types of emergency diagnosed were pulpitis, acute apical periodontitis, abscess, dental trauma, and periodontal emergencies. Pulpitis or inflammation of the dental pulp is the first complication linked to an untreated dental caries, and it is clinically diagnosed based on severe painful symptoms in a vital tooth [40]. Acute apical periodontitis can occur as a complication of dental caries and trauma, or it can be caused by iatrogenic factors and manifests as a throbbing pain that is increased by mastication or biting [41]. These two types of dental emergency were dominant in all three years. In 2019 and 2020, pulpitis predominated, followed by acute apical periodontitis, and the two constituted 73.2% of all emergencies treated in children and adolescents in 2019, and 80.8% in 2020. In 2021, acute apical periodontitis predominated, followed by pulpitis, the two pathologies constituting 76.4% of all treated dental emergencies. Al Masri et al. (2021) reported irreversible pulpitis as the most frequent diagnosis in 2019, similar to this study, but in 2020, contrary to our results, the most frequent pathology involved the gingival tissue or oral mucosa [42]. In this study, in 2020, only 8.6% of patients were diagnosed with periodontal emergencies. Other studies also showed that pulpitis and acute apical periodontitis remain among the most diagnosed oral diseases, both during the COVID-19 pandemic [43,44] and before the pandemic [45], with pain being the main reason for admittance into the dental emergency department [32].

Patients aged between 0 and 6 years were more frequently associated with emergencies caused by dental trauma, both in lockdown, pre-lockdown and post-lockdown. Odersjö et al. (2017) identified a high incidence of dental trauma in children aged between 0 and 4 years [46], and Tello et al. (2016) indicated an increasing trend in the prevalence of trauma in this age group [47]. Among the most frequent causes of trauma in this age group are falls and accidents during playtime [46]. Periodontal emergencies were significantly associated with patients in the 7–12 age group, both in 2019 and 2020. The most likely cause could be poor oral hygiene, specific to this age group [48].

We consider that the results of this research are valuable and draw attention to the high incidence of untreated dental caries among children and adolescents, and to the need for an intensive oral disease prevention program aimed at educating both children [49] and parents [50]. It is also necessary to reduce the overload of the medical staff of the dental emergency department, because psychological stress and fatigue were high among healthcare workers, especially during the COVID-19 pandemic [51].

However, this research has some limitations. Firstly, it is a retrospective study, so the data recorded in the medical files may be erroneous. Secondly, children can misreport and accentuate or diminish the intensity of a certain symptom, causing a diagnosis to be incorrect. Despite these limitations, the study provides new information, being the first study in Romania, to the best of our knowledge, that reports on the impact of the COVID-19 pandemic on the dental emergency service delivering treatments to Romanian pediatric patients during the pre-lockdown, lockdown, and post lockdown periods.

## 5. Conclusions

The number of children and adolescents treated for dental emergencies was high, with patients in the 7–12 years age group being the most affected. The most affected teeth were located in the lower and upper posterior dental arches. Patients living in the urban environment were more frequently treated than patients living in the rural environment. Pulpitis and acute apical periodontitis represent the most frequent pathologies that were diagnosed in the dental emergency department in all three years and in most age groups. The prevention of dental caries and the subsequent complications must become a priority for the Romanian dentistry.

## Figures and Tables

**Table 1:** Distribution according to age.

Age Group	2019		2020		2021	
	No.	%	No.	%	No.	%
0–6 years	57	22.1	36	18.2	24	17.6
7–12 years	123	47.9	100	50.5	59	43.4
13–18 years	77	30	62	31.3	53	39

No.—number; %—percentage.

**Table 2:** Location of the affected teeth.

	2019		2020		2021	
	No.	%	No.	%	No.	%
UA	36	14	20	10.1	16	11.8
UP	74	28.8	70	35.4	50	36.8
LA	10	3.9	3	1.5	4	2.9
LP	137	53.3	105	53	66	48.5

UA—upper anterior arch; UP—upper posterior arch; LA—lower anterior arch; LP—lower posterior arch; No.—number; %—percentage.

**Table 3:** Distribution according to the location of affected teeth and gender, age, and living environment in 2019, 2020, and 2021.

		UA	UP	LA	LP	*p* *
**2019**						
**Gender**	Girls	14	32	5	69	0.572
		(38.9%)	(43.2%)	(50%)	(50.4%)	
	Boys	22	42	5	68	
		(61.1%)	(56.8%)	(50%)	(49.6%)	
**Age**	0–6 years	19	13	2	23	0.002
		(52.8%)	(17.6%)	(20%)	(16.8%)	
	7–12 years	10	39	5	69	
		(27.8%)	(52.7%)	(50%)	(50.4%)	
	13–18 years	7	22	3	45	
		(19.4%)	(29.7%)	(30%)	(32.8%)	
**Living environment**	Rural	19	30	3	69	0.325
		(52.8%)	(40.5%)	(30%)	(50.4%)	
	Urban	17	44	7	68	
		(47.2%)	(59.5%)	(70%)	(49.6%)	
**2020**						
**Gender**	Girls	11	35	2	56	0.949
		(55%)	(50%)	(66.7%)	(53.3%)	
	Boys	9	35	1	49	
		(45%)	(50%)	(33.3%)	(46.7%)	
**Age**	0–6 years	7	12	2	15	0.029
		(35%)	(17.1%)	(66.7%)	(14.3%)	
	7–12 years	6	41	0	53	
		(30%)	(58.6%)	(0%)	(50.5%)	
	13–18 years	7	17	1	37	
		(35%)	(24.3%)	(33.3%)	(35.2%)	
**Living environment**	Rural	8	25	1	54	0.185
		(40%)	(35.7%)	(33.3%)	(51.4%)	
	Urban	12	45	2	51	
		(60%)	(64.3%)	(66.7%)	(48.6%)	
**2021**						
**Gender**	Girls	5	25	1	35	0.358
		(31.3%)	(50%)	(25%)	(53%)	
	Boys	11	25	3	31	
		(68.7%)	(50%)	(75%)	(47%)	
**Age**	0–6 years	5	9	1	9	0.707
		(31.3%)	(18%)	(25%)	(13.6%)	
	7–12 years	6	20	2	31	
		(37.4%)	(40%)	(50%)	(47%)	
	13–18 years	5	21	1	26	
		(31.3%)	(42%)	(25%)	(39.4%)	
**Living environment**	Rural	8	22	1	32	0.825
		(50%)	(44%)	(25%)	(48.5%)	
	Urban	8	28	3	34	
		(50%)	(56%)	(75%)	(51.5%)	

UA—upper anterior arch; UP—upper posterior arch; LA—lower anterior arch; LP—lower posterior arch; * Fisher’s exact test; *p* < 0.05 is statistically significant.

**Table 4:** Evolution in 2019, 2020, 2021 according to the type of emergency.

	2019		2020		2021		*p* *
	No.	%	No.	%	No.	%	
Apical periodontitis	84	32.7	73	36.9	58	42.6	0.031
Abscess	34	13.2	10	5.1	16	11.8	
Pulpitis	104	40.5	87	43.9	46	33.8	
Trauma	18	7	11	5.6	4	2.9	
Periodontal emergencies	17	6.6	17	8.6	12	8.8	

No.—number; %—percentage; * Fisher’s exact test; *p* < 0.05 is statistically significant.

**Table 5:** Distribution according to the type of emergency, gender, age, living environment in 2019, 2020, 2021.

		Apical Periodontitis	Abscess	Pulpitis	Trauma	Periodontal Emergencies	*p* *
**2019**							
**Gender**	Girls	40	11	56	5	8	0.110
		(47.6%)	(32.4%)	(53.8%)	(27.8%)	(47.1%)	
	Boys	44	23	48	13	9	
		(52.4%)	(67.6%)	(46.2%)	(72.2%)	(52.9%)	
**Age**	0–6 years	15	11	19	11	1	<0.001
		(17.9%)	(32.4%)	(18.3%)	(61.1%)	(5.9%)	
	7–12 years	42	14	48	4	15	
		(50%)	(41.2%)	(46.2%)	(22.2%)	(88.2%)	
	13–18 years	27	9	37	3	1	
		(32.1%)	(26.5%)	(35.6%)	(16.7%)	(5.9%)	
**Living environment**	Rural	43	14	51	8	5	0.506
		(51.2%)	(41.2%)	(49%)	(44.4%)	(29.4%)	
	Urban	41	20	53	10	12	
		(48.8%)	(58.8%)	(51%)	(55.6%)	(70.6%)	
**2020**							
**Gender**	Girls	35	5	46	7	11	0.713
		(47.9%)	(50%)	(52.9%)	(63.6%)	(64.7%)	
	Boys	38	5	41	4	6	
		(52.1%)	(50%)	(47.1%)	(36.4%)	(35.3%)	
**Age**	0–6 years	11	4	15	5	1	<0.001
		(15.1%)	(40%)	(17.2%)	(45.5%)	(5.9%)	
	7–12 years	46	3	33	4	14	
		(63%)	(30%)	(37.9%)	(36.4%)	(82.4%)	
	13–18 years	16	3	39	2	2	
		(21.9%)	(30%)	(44.8%)	(18.2%)	(11.8%)	
**Living environment**	Rural	27	5	49	3	4	0.025
		(37%)	(50%)	(56.3%)	(27.3%)	(23.5%)	
	Urban	46	5	38	8	13	
		(63%)	(50%)	(43.7%)	(72.7%)	(76.5%)	
**2021**							
**Gender**	Girls	30	6	25	1	4	0.467
		(51.7%)	(37.5%)	(54.3%)	(25%)	(33.3%)	
	Boys	28	10	21	3	8	
		(48.3%)	(62.5%)	(45.7%)	(75%)	(66.7%)	
**Age**	0–6 years	7	8	6	0	3	<0.001
		(12.1%)	(50%)	(13%)	(0%)	(25%)	
	7–12 years	25	7	15	3	9	
		(43.1%)	(43.8%)	(32.6%)	(75%)	(75%)	
	13–18 years	26	1	25	1	0	
		(44.8%)	(6.3%)	(54.3%)	(25%)	(0%)	
**Living environment**	Rural	29	4	26	2	2	0.042
		(50%)	(25%)	(56.5%)	(50%)	(16.7%)	
	Urban	29	12 (	20	2	10	
		(50%)	75%)	(43.5%)	(50%)	(83.3%)	

No.—number; %—percentage; * Fisher’s exact test; *p* < 0.05 is statistically significant.

## Data Availability

The data presented in this study are available on request from the corresponding authors. The data are not publicly available due to privacy reasons.
